# Case Report: Giant Paraganglioma of the Skull Base With Two Somatic Mutations in *SDHB* and *PTEN* Genes

**DOI:** 10.3389/fendo.2022.857504

**Published:** 2022-04-13

**Authors:** Ailsa Maria Main, Götz Benndorf, Ulla Feldt-Rasmussen, Kåre Fugleholm, Thomas Kistorp, Anand C. Loya, Lars Poulsgaard, Åse Krogh Rasmussen, Maria Rossing, Christine Sølling, Marianne Christina Klose

**Affiliations:** ^1^Department of Medical Endocrinology and Metabolism, Rigshospitalet, Copenhagen University Hospital, Copenhagen, Denmark; ^2^Faculty of Health and Medical Sciences, University of Copenhagen, Copenhagen, Denmark; ^3^Department of Radiology, Copenhagen University Hospital, Copenhagen, Denmark; ^4^Department of Radiology, Baylor College of Medicine, Houston, TX, United States; ^5^Department of Neurosurgery, Copenhagen University Hospital, Copenhagen, Denmark; ^6^Department of Anaesthesiology, Copenhagen University, Copenhagen, Denmark; ^7^Department of Pathology, Copenhagen University Hospital, Copenhagen, Denmark; ^8^Center for Genomic Medicine, Rigshospitalet, Copenhagen, Denmark; ^9^Department of Neuroanaesthesiology, Copenhagen University Hospital, Copenhagen, Denmark

**Keywords:** paraganglioglioma, catecholamine, neuroendochrine tumor, *SDHB* gene, alpha blockade, multidisciplinary approach, rehabilitation, Head and neck paraganglioma (HNPGL)

## Abstract

Head and neck paragangliomas (HNPGLs) are neuroendocrine tumors. They arise from the parasympathetic ganglia and can be either sporadic or due to hereditary syndromes (up to 40%). Most HNPGLs do not produce significant amounts of catecholamines. We report a case of a giant paraganglioma of the skull base with an unusually severe presentation secondary to excessive release of norepinephrine, with a good outcome considering the severity of disease. A 39-year-old Caucasian woman with no prior medical history was found unconscious and emaciated in her home. In the intensive care unit (ICU) the patient was treated for multi-organ failure with multiple complications and difficulties in stabilizing her blood pressure with values up to 246/146 mmHg. She was hospitalized in the ICU for 72 days and on the 31^st^ day clinical assessment revealed jugular foramen syndrome and paralysis of the right n. facialis. A brain MRI confirmed a right-sided tumor of the skull base of 93.553 cm^3^. Blood tests showed high amounts of normetanephrine (35.1-45.4 nmol/L, ref <1.09 nmol/L) and a tumor biopsy confirmed the diagnosis of a paraganglioma. Phenoxybenzamine and Labetalol were used in high doses ((Dibenyline^®^, 90 mg x 3 daily) and labetalol (Trandate^®^, 200 + 300 + 300 mg daily) to stabilize blood pressure. The patient underwent two tumor embolization procedures before total tumor resection on day 243. Normetanephrine and blood pressure normalized after surgery (0.77 nmol/L, ref: < 1.09 nmol/L). The damage to the cranial nerve was permanent. Our patient was comprehensively examined for germline predisposition to PPGLs, however we did not identify any causal aberrations. A somatic deletion and loss of heterozygosity (LOH) of the short arm (p) of chromosome 1 (including *SDHB*) and p of chromosome 11 was found. Analysis showed an *SDHB* (c.565T>G, p.C189G) and *PTEN* (c.834C>G, p.F278L) missense mutation in tumor DNA. The patient made a remarkable recovery except for neurological deficits after intensive multidisciplinary treatment and rehabilitation. This case demonstrates the necessity for an early tertiary center approach with a multidisciplinary expert team and highlights the efficacy of the correct treatment with alpha-blockade.

## Introduction

HNPGLs (head and neck paragangliomas) are neuroendocrine tumors. They arise from the parasympathetic ganglia and can be either sporadic or due to hereditary syndromes (up to 40%). Most HNPGLs do not produce significant amounts of catecholamines (1). Often, HNPGLs are detected in late stages due to compression or infiltration of cranial structures (1).

We report a case of a paraganglioma of the skull base with an unusually severe presentation secondary to excessive release of norepinephrine, with a good outcome considering the severity of disease after an intensive multidisciplinary approach for more than a year. Remarkably, we identified two somatic missense variants in *SDHB* (succinate dehydrogenase gene B) and *PTEN* (phosphatase and tensin homolog), respectively.

## Case Description

### Regional Medical Center

**Figure 2:**
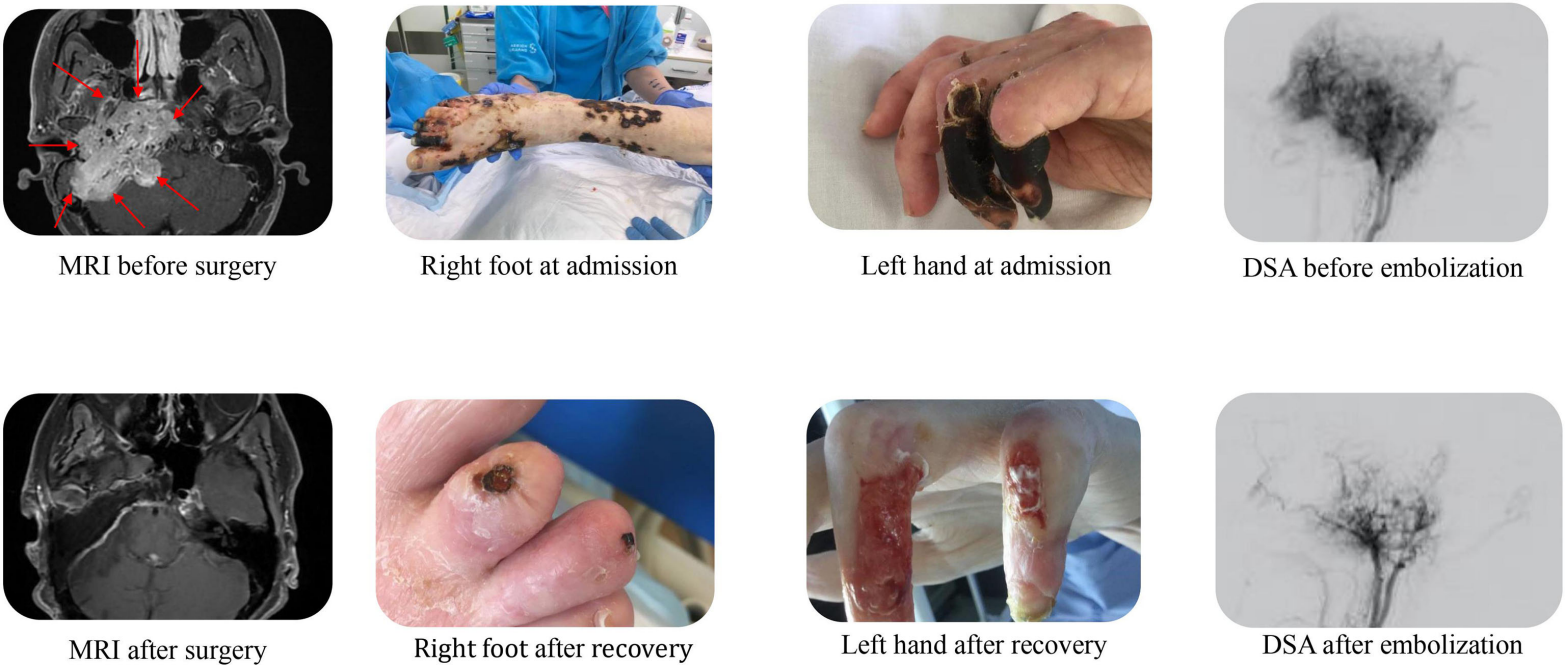
Before and after; MRI scans, pictures of scores, and DSAs. MRI (magnetic resonance imaging) scans before and after tumor resection, clinical pictures of sores on right foot and left hand at admission and at end of hospitalization, and DSA (Digital Subtraction Angiography) images before and after embolization.

A 39-year-old Caucasian woman with no prior medical history was found unresponsive by her parents in their home with a Glasgow Coma Scale (GCS) of 3. She had a respiratory rate of 5-7 per minute, hypotension, and immeasurably low blood glucose concentrations. Following a total of 500 mL 100 mg/mL intravenous glucose solution, she was admitted to the local hospital. On admission to the ICU, she had a GCS of 10, a respiratory rate of 15 per minute, blood pressure of 89/64 mmHg, pulse 94 bpm, peripheral oxygen saturation of 97%, and body temperature (ear) of 34,9° Celsius. The abdomen was swollen and tense, the skin was pale, and the extremities were bluish and marbled. Neurological examination revealed lagophthalmos on the right side and a right-sided central facial nerve palsy. No cardiac or pulmonary murmurs were present on auscultation. Cardiac ultrasonography revealed left ventricle failure with an ejection fraction of 10%. The patient had multiple chronic ulcers on her feet and lower legs, including decubitus on both heels and lower back (**Figure 2**). The patient was emaciated (38 kg, BMI: 14.8 kg/m^2^), oedematous and had severe constipation.

The paramedics described that the patient’s home showed signs of profound social deprivation with unsanitary living conditions. According to the family, she had developed a slurred speech and had been eating less than usual (mainly corn starch porridge) over the course of 2-3 months.

After the initial life-saving treatments for multi-organ failure (**Table 1**), the initial hypotension was replaced by refractory life-threatening hypertension with blood pressure spikes up to 246/146 mmHg despite treatment with carvedilol 12,5 mg x 2, ramipril 5 mg x 2, and methyldopa 500 mg x 4.

**Table 1:** Initial life-saving treatments for multi-organ failure.

Day	ICU diagnosis	Intervention
1	GCS 3on site, GCS 10 upon arrival to the ICU	CT cerebrum showing no signs of apoplexia. Basis cranii could not be evaluated due to poor imaging (artefacts due to movements)
1	Hypotension (blood pressure 89/64 mmHg)	Bolus IV fluids; 1000 mL Ringer Acetate, 100 mL human albumin, 500 mL isotonic NaCl.
1	Hypoglycaemia (blood glucose levels unmeasurably low)	500 mL 100 mg/mL glucose
1	Left ventricle failure (LVEF max 10%)	Dobutamin and norepinephrine infusions
1	Suspicion of vasculitis	75-100 mg Prednisolone daily
		Blood samples: erythrocyte sedimentation rate, immunoglobulins, anti-ANA, ANCA, ACE, beta-2- glycoprotein, HgA1c
1	ATN (Creatinine 160 um/L, eGFR 33 ml/min, Carbamide 52 mmol/l)	Haemodialysis
1	Malnourishment (BMI 14.8 kg/m^2^)	Nasogastric tube
		Thiamin, vitamin B complex, vitamin C, selenium, zinc
		Daily blood samples for refeeding syndrome
1	Suspicion of septic shock	High-dose IV antibiotics according to local protocol (meropenem, clindamycin, ciprofloxacin)
1	Suspicion of Addison’s crisis	Hydrocortisone 100 mg IV as bolus
		Hydrocortisone 200 mg IV over 24 hours
2	Blood pressure spikes up to 246/146 mmHg	Carvedilol 12.5 mg x 2, ramipril 5 mg x 2, methyldopa 500 mg x 4
2	DIC	Antithrombin III, dalteparin
	Thrombocytopenia (thrombocytes 17, ref: 145-390 x 10^9^/L)	
3	Coprostasis	Movicol (macrogol 3350 og elektrolytter), Klyx (docusat sorbitol)
4	Respiratory distress and hypercapnia	Tracheostomy and respirator treatment for a total of 49 days
4 and 15	Bilateral pleural exudates	Pleurocentesis
5	Chronic ulcers and decubitus	IV antibiotics, surgical revision, bandaging

ANA, antinuclear antibodies; ANCA, antineutrophil cytoplasmic antibodies; ATN, acute tubular necrosis; Anti-ACE, anti-angiotensin converting enzyme antibodies; DIC, disseminated intravascular coagulation; eGFR, estimated glomerular filtration rate; GCS, Glasgow Coma Scale; HgA1c, hemoglobin A1c; IV, intravenous; LVEF, left ventricular ejection fraction; SAG-M, Saline-Adenine-Glucose-Mannitol.

Respiratory distress, and hypercapnia, as well as bilateral pleural effusion ensued, necessitating intubation for 49 days and pleural drainage. Further necrotic sores evolved on her toes and fingertips. This was initially thought to be due to sepsis and DIC and amputation was considered. Aggressive treatment with antibiotics, surgical revision, and bandaging was carried out.

The patient also had stomach pain due to coprostasis, which was difficult to treat with normal laxatives.

On the 7^th^ day in the ICU, an occupational therapist described right-sided facial nerve palsy, lagophthalmos of the right eye, her uvula was pulled to the left, and she had soft palate dysfunction. Right-sided jugular foramen syndrome (paresis of the glossopharyngeal, vagal, hypoglossal, and accessory nerves) as well as a right-sided facial nerve palsy were diagnosed on day 31 following an ENT assessment.

A brain CT was performed at admission to rule out intracranial bleeding. Unfortunately, due to movement artifacts, the skull base could not be evaluated in this CT and a new brain CT was not prioritized as intracranial bleeding had been ruled out. Due to the central facial nerve palsy diagnosed on day 31 repeat CT scanning as well as magnetic resonance (MR) imaging were performed, revealing a giant (93.553cm^3^) tumor of the skull base located both intra- and extracranially (**Figure 2**). Subsequent ^18^F-FDG PET/CT imaging revealed a high FDG uptake confined to the tumor. A tumor biopsy was performed to determine the type of tumor. This showed a classic paraganglioma with a Ki-67 index of 4%. Plasma metanephrines were measured after the pathology results and showed very high concentrations of normetanephrine (35.1-45.4 nmol/L, ref <1.09 nmol/L), confirming the clinical suspicion of a catecholamine secreting paraganglioma. Chromogranin A measured on day 40 was 242 uL/L (ref: <102 uL/L).

### Tertiary Medical Center

**Figure 1:**
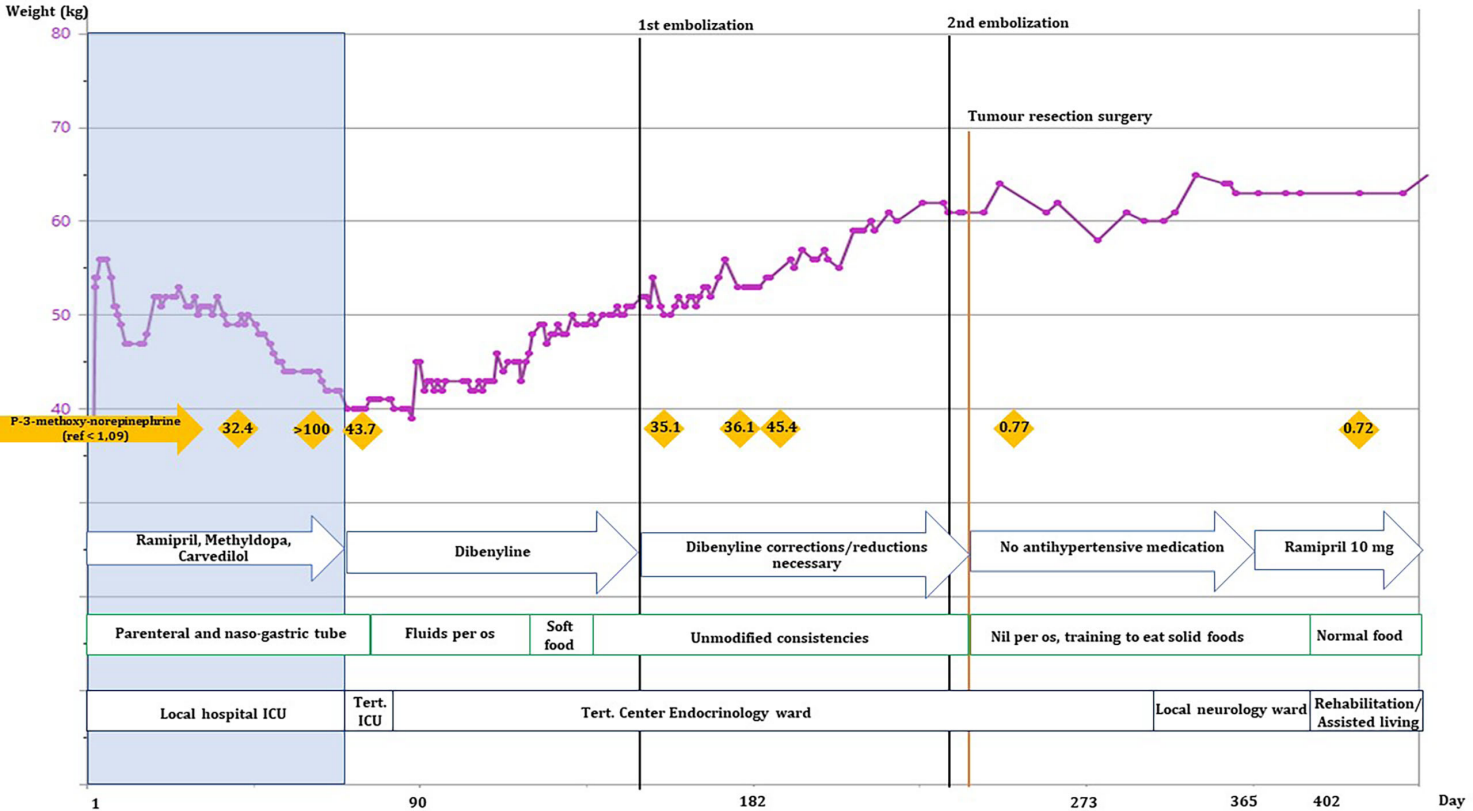
Development of the patient’s weight (kg), normetanephrine levels, antihypertensive medicine, nutritional intake, and physical location over the first 365 days of hospitalization. Black lines: embolization procedures, orange line: tumor resection surgery, orange rhombes: plasma-3-methoxy-norepinephrine levels (nmol/L), blue arrows: changes in blood pressure medications, green blocks: changes in the patient’s ability to drink/eat. She was at no point able to sustain nutrient intake without supplemental nutrition by naso-gastric tube. Black boxes show the patient’s location.

The patient spent 72 days in the local ICU before being admitted to a tertiary referral center under a multidisciplinary team. ^123^MIBG-scintigraphy showed no uptake in the tumor or elsewhere and alpha-blockade treatment was initiated. ^18^Ga-Dotatoc PET-CT showed localized grade 2 uptake in the tumor with no radiological signs of metastatic disease in the thorax or abdomen. Alpha-blockade treatment was gradually increased, according to daily orthostatic blood pressure measurements, to high doses of phenoxybenzamine (Dibenyline^®^, 90 mg x 3 daily) and labetalol (Trandate^®^, 200 + 300 + 300 mg daily). The goal was to stabilize the blood pressure and improve the general physical state before the first tumor embolization treatment. Upon alpha blockade and blood pressure stabilization the patient gained weight (**Figure 1**), and the chronic sores and necrotic fingertips and toes started to heal (**Figure 2**).

The patient’s nutrient intake was closely regulated by a dietician to help her gain weight together with close monitoring of blood samples to avoid refeeding syndrome. Together with physio- and occupational therapists, ADL (activities of daily living) functions were assessed and trained intensively. After approximately 2 months of alpha-blockade treatment the patient was able to swallow bread crusts, not just liquids and soft foods (see **Figure 1**).

Finally, the patient was physically strong enough and the first tumor embolization was performed on day 150. Tumor branches of the ascending pharyngeal artery were embolized with Embospheres and an anastomosis was coiled. This reduced the anterior spinal artery supply to about 60-70%. Between the first and the second tumor embolization the patient’s antihypertensive medication was adjusted to phenoxybenzamine 70 + 80 + 80 mg daily and labetalol 300mg x 3 daily. Before the second embolization on day 238 an MRI scan showed tumor growth since the first embolization (6 x 5.4 x 5.5 cm). The right internal carotid artery was coiled, and the anterior tumor compartment was subsequently embolized. Complete embolization of all the arterial tumor supply was not achieved.

In relation to the surgical procedures the patient was categorized as an ASA (American Society of Anesthesiologists) score of 4, indicating an immediately life-threatening condition. Preparations in the form of an arterial line for continuous blood pressure monitoring and large bore central venous catheter was inserted as moderate to severe blood loss was expected.

Phenoxybenzamine and labetalol were discontinued on the day of the craniotomy on day 243 when the tumor was resected by a total petrosectomy. Infusions with adenosine and magnesium-sulphate were started at the induction of anesthesia. Intravenous norepinephrine was infused intermittently to maintain mean arterial blood pressure (MAP) between 70-100 mmHg. In relation to the petrosectomy a defect in the dura in the posterior fossa was repaired with pericranium. Fat tissue from the abdomen was harvested to fill the petrous bone defect and the defect was restored by a titanium cranioplasty. The procedure lasted close to seven hours and the hemorrhage volume (estimated at 5100 ml) was replaced with packed red blood cells, plasma, and platelets in accordance with local guidelines and thromboelastographic testing of coagulation. Respiratory and circulatory stability was achieved during all three procedures in general anesthesia. Infusions with adenosine, magnesium and norepinephrine were adjusted frequently in the 2 days after surgery to keep a MAP above 80 and systolic blood pressure below 180 mmHg.

Normetanephrine normalized after surgery (0.77 nmol/L, ref: < 1.09 nmol/L) and her blood pressure normalized (approx. 130/80 mmHg) without antihypertensive drugs. The damage to the cranial nerves (n. glossopharyngeus, n. vagus, n. hypoglossus, n. accessories, and n. facialis) was permanent.

#### Pathology, Genetic Analysis, and Diagnosis

The tumor cells stained positive for synaptophysin, chromogranin, and S-100 along with SOX10 highlighted haphazardly distributed sustentacular cells. SDHA immunostaining was strongly positive, however *SDHB* was faint and thus difficult to conclude upon. The pathology analysis of the excised tumor itself showed the same histopathological patterns. Also, staining revealed loss of *PTEN* staining and alpha-inhibin staining was focally and weekly positive in tumor cells.

Molecular analysis including sequencing on tumor DNA revealed an *SDHB* (c.565T>G, p.C189G) and *PTEN* (c.834C>G, p.F278L) missense mutation. An SNP-array of tumor DNA showed loss of heterozygosity of p.1 (including *SDHB*) and p.11 in tumor tissue. Germline DNA was sequenced in parallel and the initial gene panel analysis did not show any variants in *FH, MAX, MEN1, NF1, RET, SDHX, THEM127* or *VHL.* Subsequent whole genome sequencing on germline DNA did not reveal any genetic etiology of the disease.

### Follow-Up and Outcome

The patient was transferred to a local neurology ward on day 292. The primary focus was to improve Activity of Daily Life with physio- and occupational therapists and after a total of 402 days in hospital she was transferred to a rehabilitation facility. She has been fitted with a Montgomery prosthesis to help her weak voice and may in future regain the ability to eat normal foods. Follow-up MRI three months post-surgery showed no signs of tumor remnant or regrowth. Future lifelong follow-up is planned according to international guidelines for pheochromocytomas and paragangliomas (PPGLs) (2).

## Discussion

This case of a giant paraganglioma of the skull base with an unusually severe presentation secondary to excessive release of norepinephrine highlights how the correct diagnosis, treatment, and the use of a multi-disciplinary approach can result in a relatively good outcome.

Our patient presented with a multi-organ crisis which is a rare presentation in PPGLs. She developed pulmonary exudates, acute renal failure and DIC. The symptoms were at first confused with sepsis, which has also previously been reported in other severe cases (3). PPGLs can present with life-threatening cardiovascular manifestations (3, 4). In our case the cardiogenic shock was most likely associated with severe hypotension on the basis of pump failure due to a severe left ventricular dysfunction, which has previously been reported in a substantial number of patients with PPGLs (4, 5).

HNPGLs that secrete catecholamines give rise to hypertension and in our case the norepinephrine-mediated alpha receptor stimulation resulted in vasoconstriction and hypertension (6) but also episodes of orthostatic hypotension. It has previously been reported that norepinephrine can cause orthostatic hypotension. The mechanisms behind this could be an impaired vasoconstrictor response due to downregulation and desensitization of the alpha-adrenergic receptors and feedback inhibition of sympathoneuronal norepinephrine release either by sympathoinhibition or as a result of stimulation of presynaptic alpha2-adrenergic receptors (3, 7). It is however difficult to discern whether the patients cachectic state could also have contributed to or be the reason for the orthostatic hypotension.

Our patient also suffered from peripheral ischemia with necrosis due to extreme vasoconstriction caused by hypercatecholaminaemia which has previously been reported in other patients with extremely high concentrations of normetanephrine (6).

Together with long term immobilization the excess amount of catecholamines could explain the coprostasis. Norepinephrine inhibits the plexus of the enteric nervous system which will inhibit bowl movements, gastrointestinal secretions and blood flow. Also, norepinephrine inhibits the presynaptic release of acetylcholine which stimulates normal bowel functions (8). This can explain why it was so difficult to treat the constipation, which is a rare but previously described complications in PPGLs (8).

The question is still how the patient managed to develop such a severe disease with pronounced disease manifestations due to many months of tumor pressure and elevated catecholamine levels. In our case the suspicion is that the predominant cause is severe social deprivation, but it has not been possible to obtain more detailed information about the patient’s life or physical health in the time leading up to admission. After admission to the local ICU it took more than a month before the tumor was discovered as the underlying cause. Unfortunately, the tumor was not diagnosed at the initial CT scan on the day of admission due to artefacts on the scan performed mainly to rule out intracranial hemorrhage. The right facial nerve palsy, the lagophthalmos of the right eye and other signs of foramen jugulare syndrome were described both at admission and on day 7 following her admission. It is unclear why this was not followed up immediately but first on day 31 at the ENT assessment. The symptoms may have been overlooked due to the multiple other life-threatening symptoms. Had the central nerve palsy been diagnosed properly at an earlier stage a repeat CT of the brain would have revealed the tumor at the skull base.

The differential diagnosis of a HNPGL had not been considered prior to the biopsy, and the patient thus underwent a tumor biopsy due to suspicion of a malignant brain tumor. Had a HNPGL been considered as differential diagnosis, then the appropriate succession of events would have been to measure the catecholamines prior to taking the biopsy to ensure relevant alpha-blockade treatment to minimize the risk for the patient (9). This would also have provided the possibility of measuring the dopamine metabolite 3-methoxytyramine which is a very strong and relevant predictor of metastases development (10) in a patient with a somatic *SDHB* mutation.

After the initial CT and MRI scan our patient underwent ^18^Ga-Dotatoc PET-CT which is first choice in HNPGL detection (9, 11).

The patient’s poor physical state protracted the progress and it took many months to get her physically strong enough to undergo the tumor resection. The correct tumor diagnosis proved detrimental to improving the patient’s physical strength as this clearly required the correct treatment with alpha-blockade. In our case the patient’s weight stabilized just after the alpha-blockade treatment was initialized (**Figure 1**). Subsequently she gained weight at a steady rate and was back to her normal weight (approximately 60 kg, BMI: 23.4 kg/m^2^) on day 214. The initial weight stabilization and weight gain could be attributed to the alpha-blockade treatment as the excess catecholamines are known to increase metabolism in patients, resulting in weight loss (12, 13). After approximately 2 months of alpha-blockade the patient regained the ability to swallow more solid foods which likely contributed to her further weight gain. It has previously been shown in a study on rats that catecholamines can inhibit the swallowing reflex (14).

Patients with catecholamine producing PPGLs have an increased risk of developing diabetes (13). This is mainly caused by high levels of circulating catecholamines leading to compromised insulin secretion from the pancreatic β-cells, decreased glucose uptake, and increased insulin resistance (15). Perhaps, did not occur in our patient (HbA1c 38-40 nnmol/mol (ref <48 nmol/mol)) because of her emaciated state and lack of food intake at admission where she was severely hypoglycemic due to her cachectic and catabolic condition. One could also speculate, that the reason for her not developing diabetes during her hospital stay was because of the alpha-blockade treatment.

A previous case report from 2011 concluded, that multidisciplinary approach is necessary when treating large secreting intracranial paragangliomas. Mainly, the use of pre-operative embolization decreases the risk of surgical hemorrhage before complete surgical resection which is the optimal treatment as a curative aim (16). The embolization approach has also been used in other casuistic cases (17) and a systematic review and meta-analysis concluded that embolization of carotid body tumors prior to surgery reduced blood loss during surgery (18).

Our patient was comprehensively examined for germline predisposition to pheochromocytomas and paragangliomas, however we did not identify any causal aberrations. Rare cases of sporadic paragangliomas due to somatic variants of *SDHB* have been reported (19–21). Pathology immunohistochemistry analysis showed loss of *PTEN* expression in tumor tissue, underlining the influence of the *PTEN* missense mutation. Our case is the first to report highlight the possible significance of *PTEN* mutations in PPGLs. *PTEN* is one of the most frequently somatically mutated genes in cancer (22–24) and has been reported in brain tumours, lung cancer, prostate cancer, endometrial cancer breast cancer and pancreatic cancer (22). Furthermore, a quarter of thyroid adenomas and several sporadic malignant thyroid cancers were found to have *PTEN* LOH (22).

The somatic *PTEN* variant (c.834C>G, p.F278L) has previously been reported, but not in PPGLs (25). The *SDHB* variant (c.565T>G, p.C189G) has not previously been reported and neither variant is annotated in dbSNP (https://www.ncbi.nlm.nih.gov/snp/), ClinVar (https://www.ncbi.nlm.nih.gov/clinvar/), or in GnomAD (https://gnomad.broadinstitute.org/). *PTEN* mutations have not been found in human PPGLs but LOH at the *PTEN* loci has previously been reported in 2% (26) to 16% of examined tumors (27) Furthermore it has been shown in studies on mice that *PTEN* mutations can cause development of malignant PPGLs (27) and the loss of *PTEN* expression has therefore been associated with the progression of these tumors.

Alpha-inhibin immunohistochemistry was focally and weekly positive in tumor tissue. The expression of alpha-inhibin has been suggested as a screening tool for pseudo-hypoxic paragangliomas (Cluster 1 disease including *SDHB*-related disease) (28). Although alpha-inhibin cannot stand alone as a diagnostic tool for *SDHx*-related disease (28) it may contribute in cases where the *SDHB* expression is difficult to conclude on as in our case.

*SDHB* mutations in general are strongly associated with recurrence (29). Furthermore, as per the newest WHO guidelines, paragangliomas are not differentiated into benign or malignant as these tumors are all potentially malignant and should have follow-up according to guidelines (30).

The TIER classification system is a proposed alternative for classifying somatic variants (31). Even though we suspect that this variant of *SDHB* could be pathogenic we have not been able to classify it as more than a TIER III class variant (Variant of Unknown Significance) based on the lack of functional studies. Based on our current knowledge it is therefore not possible to predict the future clinical outcome for our patient and she will be followed with lifelong clinical screenings.

In conclusion we describe a case with an unusually severe presentation of a giant skull base PPGL with a relatively good outcome under the given circumstances. This case demonstrates the necessity for an early tertiary center approach with multidisciplinary expert teams. Furthermore, it highlights the efficacy of the correct treatment with alpha-blockade sooner rather than later. The condition is treatable but can be fatal and have serious long-term consequences for the patients. There is need for a more extensive knowledge on how to follow up on patients with PPGLs based on genetic, pathological, and clinical symptoms in the individual patient.

## Data Availability Statement

The original contributions presented in the study are included in the article/supplementary material. Further inquiries can be directed to the corresponding author.

## Ethics Statement

Written informed consent was obtained from the individual(s) for the publication of any potentially identifiable images or data included in this article.

## Author Contributions

AM: first author, data collection, manuscript writing, and manuscript editing. GB: expert on tumor embolization procedure, manuscript proofreading, and writing section on embolization procedure. UF-R: expert in treatment of catecholamine producing tumors pre-op, writing section on endocrine treatment, and manuscript proofreading. KF: expert in neurosurgery, manuscript proofreading, and writing on section on perioperative procedure. TK: expert in anesthesiology and writing on section on section on perioperative procedure. AL: expert in pathology and writing section on pathology findings. LP: expert in neurosurgery and writing on section on neuroimaging. ÅR: expert in treatment of catecholamine producing tumors pre-op, writing section on endocrine treatment, and manuscript proofreading. MR: expert in genetic analysis and writing section on genetic analysis finding. CS: expert in anesthesiology and writing on section on perioperative procedure. MK: expert in treatment of catecholamine producing tumors pre-op, writing section on endocrine treatment, and manuscript proofreading. All authors contributed to the article and approved the submitted version.

## Funding

UF-R’s research salary is sponsored by The Kirsten and Freddy Johansen’s Fund.

## Conflict of Interest

The authors declare that the research was conducted in the absence of any commercial or financial relationships that could be construed as a potential conflict of interest.

## Publisher’s Note

All claims expressed in this article are solely those of the authors and do not necessarily represent those of their affiliated organizations, or those of the publisher, the editors and the reviewers. Any product that may be evaluated in this article, or claim that may be made by its manufacturer, is not guaranteed or endorsed by the publisher.
